# Evaluating the biological relevance of the relativistic theory of consciousness

**DOI:** 10.3389/fpsyg.2026.1877478

**Published:** 2026-08-07

**Authors:** Wolfgang Kromer

**Affiliations:** Retired, Konstanz, Germany

**Keywords:** comparator model, consciousness, explanatory gap, hard problem of consciousness, relativistic theory of consciousness, self-awareness

## The subject of discussion

The Relativistic Theory of Consciousness by Lahav and Neemeh (2022) offers an interesting and thought-provoking, new view on consciousness. However, it also raises questions about the underlying concept of consciousness and about its real-world applicability and, hence, its biological relevance. The mathematical formulation of the theory will not be a matter of discussion because it provides just a theoretical concept but does not contribute any useful information concerning the open issues mentioned above.

The theory states that consciousness is not an absolute feature but a relativistic one, that depends on the reference system of the observer. Accordingly, the first-person frame of reference (the frame of reference of the cognitive system) doesn't measure physical (neural) patterns, but actually “what they represent and stand for. For example, the representation of an apple has some causal power in the system [...] and will trigger [...] memories, emotions, and motor processes. For the cognitive system, this is what it means to be an apple.” (Lahav and Neemeh, 2022). And further: “From outside [...], as in the position the neuroscientist takes as a third-person observer, the same exact phenomena appear as neural computations.” In other words, both perspectives “describe the same underlying reality.” The Relativistic Theory of Consciousness thereby claims to solve the “hard problem of consciousness,” (Chalmers, 1995), i.e., the question of how to bridge the “explanatory gap” between physical (neuronal) processes and their subjective, phenomenal correlates (feelings, emotions). Lahav and Neemeh (2022) summarize: “Phenomenal consciousness is neither private nor delusional, just relativistic.”

This raises two critical issues. First: What is the fundamental precondition of consciousness which the Relativistic Theory may be based upon? Although this aspect does neither question the relativistic principle as such nor its biological relevance, it nonetheless refers to the theory's basic assumptions and should therefore be addressed first. To this end, a supplementary model of consciousness will be introduced.

Second: What is the theory's biological relevance? This solely depends on the theory's real-life applicability because this is it what the authors fundamentally envisage when saying “all we need to do to measure phenomenal properties is to change our perspective by moving to the appropriate frame of reference.” Thus, the technological requirements for this transition are a most critical issue. When based on suitable premises, the mathematical formulation of changing from a third-person to a first-person frame of reference is of course possible and may well be internally consistent, but this does not *per se* prove any corresponding biological reality.

## The underlying concept of consciousness

Lahav and Neemeh (2022) note that “there are still several open questions that need to be addressed in the future. For example, what are the necessary and sufficient conditions for a cognitive frame of reference to have self-consciousness and, consequently, phenomenal consciousness?” Thus, the underlying, fundamental concept of self-consciousness is obviously an unanswered issue in the Relativistic Theory of Consciousness.

Actually, there are numerous publications that describe diverse neuronal mechanisms involved in the generation of conscious percepts (for references, see: Maia and Cleeremans, 2005; Mashour et al., 2020; Ciaunica et al., 2021). Nevertheless, the title of an editorial by Aru and Bachmann (2015) is valid even today: “Still wanted – the mechanisms of consciousness!” The reason is that the essential aspect hasn't been adequately considered yet. Many authors (for example: Prinz, 2009; Edelman et al., 2011; Newen, 2018; Riva, 2018) have emphasized embodiment as a significant feature of consciousness, but did not arrive at a final conclusion about the neuronal function that provides the ultimate basis for an embodied self and, consequently, self-consciousness.

A comparator model (compare to: Johnson et al., 1993; Zaadnoordijk et al., 2019) might answer this question best. Actually, consciousness (in the sense of being aware of one's existence) requires an individual to whom something can become conscious. However, only if the organism can distinguish between its own body and the environment by comparatively processing internal vs. external stimuli and their resulting sensations, the organism can experience itself as an individual as opposed to the outside world. Only then can consciousness be allocated. Thus, the experience of self results from the comparative representation of bodily vs. environmental sensations (Kromer, 2022, 2025, 2026), irrespective of the underlying (no doubt extremely complex) neuronal mechanisms. Naturalists regard this phenomenon of self-experience as an “illusion” but, more rationally, it may simply be regarded as a comparison image composed of complex contributions from inside vs. outside one's own body. Actually, neuronal networks can (for example) facilitate or weaken impulse transmission thereby performing filter functions, they can encode information and store engrams for memory, and they can keep attention (at least to some extent) simultaneously on information from inside and outside the own body (“divided attention”; Kim et al., 2017; Zhang et al., 2017). Thereby sensory signals from the two sources are constantly represented in comparison to each other. But neurosciences do not provide any evidence that neuronal networks can accomplish anything principally beyond those functions.

**Figure 1:**
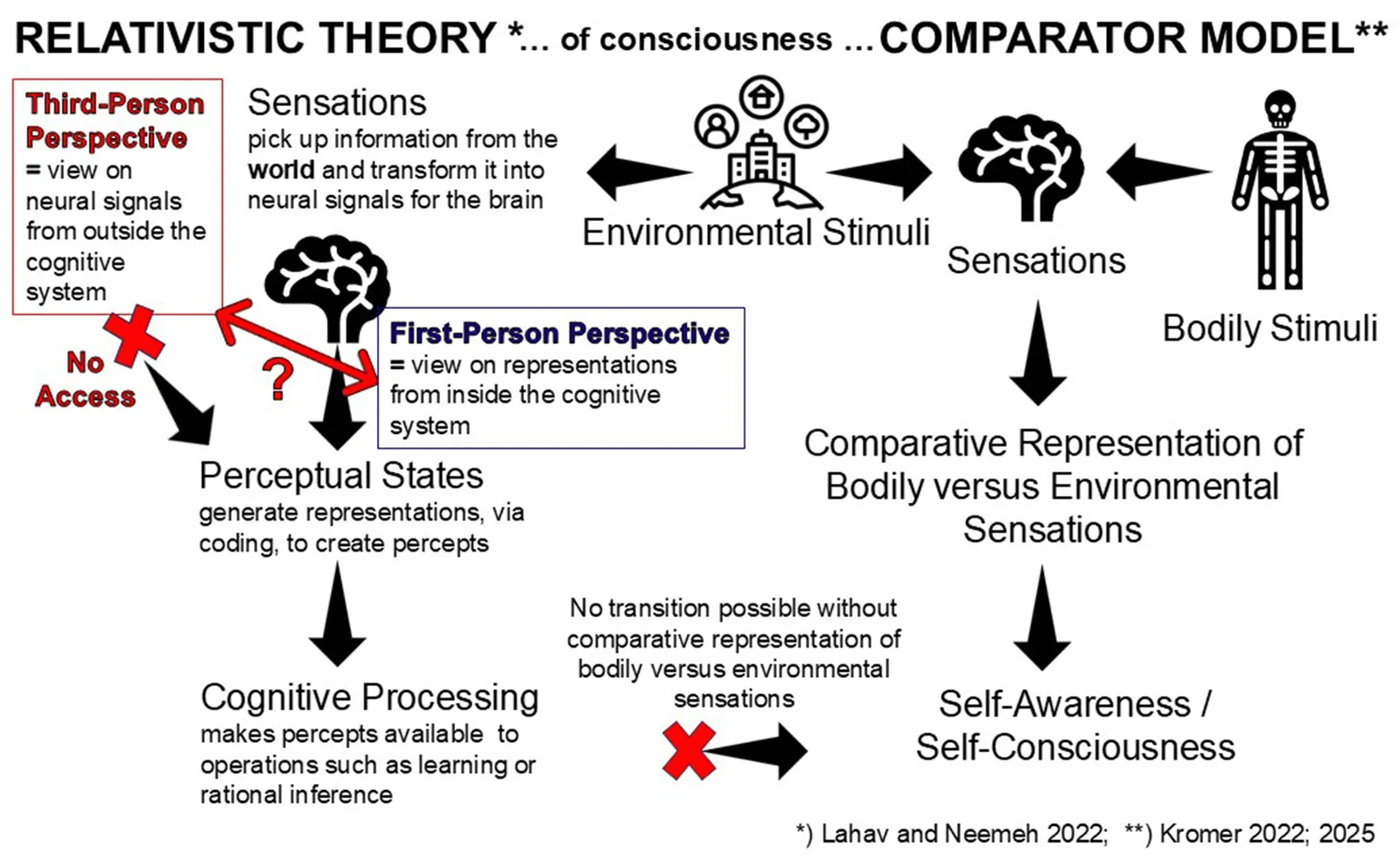
Visualization of the relativistic theory of consciousness by Lahav and Neemeh (2022; left) and the comparator model of consciousness by Kromer (2022, 2025; right). The figure depicts selected aspects of the two theories. Top-left corner: The third-person frame of reference does not provide access to perceptual states. The double-headed arrow indicates a hypothetical transformation from one frame of reference to the other. The question mark below the double-headed arrow refers to the lack of technological tools required to carry out such a transformation. Bottom, between the two models: According to the comparator model, transition from cognitive processing to self-consciousness is blocked because the Relativistic Theory does not consider comparative representation of bodily vs. environmental sensations. (Free pictograms taken from PowerPoint, Microsoft).

Lahav and Neemeh (2022) do not address the crucial aspect of embodiment but instead only note that a “sensation picks up information from the world and transforms it [...] into neural signals for the brain.” It remains therefore open if, and how, the aspect of embodiment would affect the concept of Lahav and Neemeh. Figure 1 depicts essential aspects of the Relativistic Theory of Consciousness in comparison to the Comparator Model (Kromer, 2022, 2025) and draws the attention to one crucial point: Is the emergence of self-consciousness conceivable although embodiment has not been taken into account? (see legend for details). Without prejudice to this question, both the relativistic theory and the comparator model arrive, although based on different conceptual foundations, at the conclusion that the “explanatory gap” is an illusory one.

## Real-world applicability and biological relevance

The essential point that Lahav and Neemeh (2022) make is: “All we need to do to measure phenomenal properties is to change our perspective by moving to the appropriate frame of reference, i.e., the frame wherein the representations have causal power.” Thus, it's a matter of “the right kind of measurement.” But Lahav and Neemeh also note that “no such transformation is currently technologically feasible, but it is nomologically possible” (compare to Figure 1: the double-headed arrow with the question mark). And they further note: Because “we lack the accurate equations of the cognitive system [...] the transformation equation will also be in a general form.”

At this stage, the Relativistic Theory of Consciousness certainly appears to be mathematically well formulated in analogy to the principle of relativity as known from physics, but its biological relevance remains totally obscure. Actually, this would require the theory's applicability in real life and therefore the ability to somehow read out the neural pattern of another person in order to then change to that person's first-person frame of reference. Is it this futuristic vision that the authors have in mind? They don't comment on that specifically but at least note that “no such transformation [from the third-person to the first-person frame of reference] is currently technologically feasible,” thereby implying that it may *some day* become feasible.

But what might be the technological tools to get hold of another person's extremely complex, neural pattern? And once this task should have been solved, once again suitable technological tools would be required to incorporate the observed person's neural pattern into the cognitive system of the observer in order to get him access to the observed person's phenomenal experience. Still then, the observer would not be automatically in the first-person frame of reference of the observed person. That is because in the living brain, in contrast to a computer, the hardware is identical with the software (Laydevant et al., 2024) and of course differs among individuals.

Only if all that matches (by chance?) between the observer and the observed person, an unrestricted neural pattern might implement the dynamics to lay the foundation for the respective, phenomenal experience. Actually, the dynamics of the cognitive system are defined by the complex integration of all parts of the central nervous system that specifically contribute to a phenomenal experience, including all the engrams that originally contributed to the observed person's phenomenal experience. What for would then a “transformation equation” be required, and how would it be applied in a real-world scenario? Moreover, once the observed person's neural pattern has been (theoretically) successfully incorporated into the cognitive system of the observer, it will there face the observer's intimate biographical engrams which may affect the resulting phenomenal experience. Pure theory might ignore these interactions for the sake of simplicity, biology will not. On the other hand, applying a transformation equation to an extracted neural pattern on a computer (however the computer program might implement the neural pattern) would not end up in any phenomenal experience. Who should have it, then?

Specific problems may also arise when considering the biological variation in neuronal functions. For example, sensory stimuli triggered by a fire that produced temperature x and specific wavelengths of light y, might be “depicted” by neurons of person A as x(A) and y(A), but by those of person B as somewhat different measurements x(B) and y(B). Those differences may not be mirrored by differences in the dynamics (i.e., the complex weave of interconnections) but may nevertheless be reflected by the individual, phenomenal experience. How would that be represented and in practice implemented by any (still not “accurate”) transformation equation?

However one might look at this scenario, it may turn out to be futuristic vision. “Non-invasive brain recordings” (Tang et al., 2023) are far removed from the tools required in the present context. No solution to the problem of real-world transformation from one frame of reference to the other is in sight, not even faintly. Although it had been wildly speculated in discussion groups that future technology might allow to read out the subjective experience of other persons, the technological obstacles in real life are likely to be enormous, and the problem will probably never be solved.

Should this futuristic project in fact fail, the Relativistic Theory of Consciousness would remain purely academic. These technological difficulties may not invalidate the formal correctness of the relativistic principle outlined by Lahav and Neemeh (2022), but they will prevent its application in real life and thereby its biological relevance. Nevertheless, as a conceptual system, the Relativistic Theory of Consciousness may appear attractive as it avoids the conflicting views offered by dualists vs. illusionists. Lahav and Neemeh (2022) note: “If we can argue *against* the privacy of phenomenal properties, then we can escape the trap into which both the dualist and illusionist fall,” i.e., “the hard problem” of consciousness (Chalmers, 1995). According to Lahav and Neemeh (2022), “the hard problem dissolves” because of the relativistic nature of consciousness. With respect to the “hard problem of consciousness” (and only in this respect), the Relativistic Theory of Consciousness meets the Comparator Model (Kromer, 2022, 2025), although based on completely different reasoning.

## Conclusion

The Relativistic Theory of Consciousness offers a thought-provoking, but purely formal analogy to the principle of relativity as known from physics. The authors' basic statement that the first-person perspective (phenomenal experience) and the third-person perspective (observed neural patterns) describe the same underlying reality appears to be a reasonable and even obvious conclusion. Nevertheless, the implied real-world transformation from a third-person to a first-person frame of reference and, hence, the theory's biological relevance remain highly obscure. Should this task most probably fail in the future, the theory will remain purely formalistic but may nevertheless appear internally consistent. However, any proof of the theory's concept of getting access to another person's phenomenal experience by changing the frame of reference will require real-world practicability because any phenomenal experience is restricted to the individual. It will happen neither on paper nor on a computer. Apart from that, and in agreement with a Comparator Model based on a completely different reasoning, the theory states that the “hard problem of consciousness” disappears and that there is no longer any explanatory gap. This makes the theory at least intellectually attractive.
